# Chemistry and Pharmacokinetics of Gallium Maltolate, a Compound With High Oral Gallium Bioavailability

**DOI:** 10.1155/MBD.2000.33

**Published:** 2000

**Authors:** Lawrence R. Bernstein, Trevor Tanner, Claire Godfrey, Bruce Noll

**Affiliations:** 1 GeoMed, Inc., 285 Willow Road Menlo Park CA 94025 USA; 2 SlMBEC Research Ltd. Merthyr Tydfil CF48 4DR United Kingdom; 3 Department of Chemistry and Biochemistry University of Colorado Boulder CO 80309 USA

## Abstract

Gallium maltolate, tris(3-hydroxy-2-methyl-*4H*-pyran-4-onato)gallium (GaM), is an orally active gallium compound for therapeutic use. It is moderately soluble in water (10.7 ± 0.9 mg/mL at 25∘C) with an octanol partition coefficient of 0.41±0.08. The molecule is electrically neutral in aqueous solution at neutral pH; a dilute aqueous solution (2.5 ×10−^-5^  M) showed little dissociation at pH 5.5-8.0. Single crystal X-ray diffraction analysis found the GaM molecule to consist of three maltolate ligands bidentately bound to a central gallium atom in a propeller-like arrangement, with one of the ligands disordered in two possible orientations. The compound is orthorhombic, space group *Pbca*, unit cell *a* = 16.675(3), *b* = 12.034(2), *c* = 18.435(2) Å at 158K. GaM was administered to healthy human volunteers at single doses of 100, 200, 300, and 500 mg (three subjects per dose). GaM was very well tolerated. Oral absorption of Ga into plasma was fairly rapid (absorption half life = 0.8-2.0h), with a central compartment excretion half life of 17-21h. Absorption appeared dose proportional over the dosage range studied. Estimated oral gallium bioavailability was approximately 25-57%, based on comparison with published data on intravenous gallium nitrate. Urinary Ga excretion following oral GaM administration was approximately 2% of the administered dose over 72h, in contrast to 49-94% urinary Ga excretion over 24h following i.v. gallium nitrate administration. We suggest that oral administration of GaM results in nearly all plasma gallium being bound to transferrin, whereas i.v. administration of gallium nitrate results in formation of considerable plasma gallate [Ga(OH)_4_^−^], which is rapidly excreted in the urine. These data support the continued investigation of GaM as an orally active therapeutic gallium compound.


### CHEMISTRY AND PHARMACOKINETICS OF GALLIUM MALTOLATE, A COMPOUND WITH HIGH ORAL GALLIUM BIOAVAILABILITY

Lawrence R. Bernstein[1*], Trevor Tanner[2], Claire Godfrey[2] and Bruce Noll[3]

[1]GeoMed, Inc., 285 Willow Road, Menlo Park, CA 94025 USA
[2]SIMBEC Research Ltd., Merthyr Tydfil, CF48 4DR, United Kingdom
[3]Department of Chemistry and Biochemistry, University of Colorado, Boulder CO 80309 USA

### ABSTRACT

Gallium maltolate, tris(3-hydroxy-2-methyl-4*H*-pyran-4-onato)gallium (GaM), is an orally active gallium compound for therapeutic use. It is moderately soluble in water (10.7±0.9 mg/mL at 25°C) with an octanol partition coefficient of 0.41±0.08. The molecule is electrically neutral in aqueous solution at neutral pH; a dilute aqueous solution ($2.5\times10^{-5}$ M) showed little dissociation at pH 5.5-8.0. Single crystal X-ray diffraction analysis found the GaM molecule to consist of three maltolate ligands bidentately bound to a central gallium atom in a propeller-like arrangement, with one of the ligands disordered in two possible orientations. The compound is orthorhombic, space group *Pbca*, unit cell $a$ = 16.675(3), $b$ = 12.034(2), $c$ = 18.435(2) Å at 158K. GaM was administered to healthy human volunteers at single doses of 100, 200, 300, and 500 mg (three subjects per dose). GaM was very well tolerated. Oral absorption of Ga into plasma was fairly rapid (absorption half life = 0.8-2.0h), with a central compartment excretion half life of 17-21h. Absorption appeared dose proportional over the dosage range studied. Estimated oral gallium bioavailability was approximately 25-57%, based on comparison with published data on intravenous gallium nitrate. Urinary Ga excretion following oral GaM administration was approximately 2% of the administered dose over 72h, in contrast to 49-94% urinary Ga excretion over 24h following i.v. gallium nitrate administration. We suggest that oral administration of GaM results in nearly all plasma gallium being bound to transferrin, whereas i.v. administration of gallium nitrate results in formation of considerable plasma gallate [$Ga(OH)_4^-$], which is rapidly excreted in the urine. These data support the continued investigation of GaM as an orally active therapeutic gallium compound.

### INTRODUCTION

Gallium is a semi-metallic element that has shown therapeutic activity in metabolic bone disease, hypercalcemia and cancer [1]. It is approved for use in the United States as citrate-chelated gallium nitrate solution for intravenous infusion to treat hypercalcemia of malignancy [2]. Gallium reduces plasma calcium by inhibiting bone resorption, predominately through selective actions on osteoclasts and osteoblasts [1]. Clinical studies have shown that gallium administered intravenously or subcutaneously can reduce the abnormally high turnover of bone characteristic of Paget's disease [3] and can prevent osteolysis, ameliorate pain, and extend survival in patients with multiple myeloma [4,5]. Gallium has also been suggested as a possible treatment for osteoporosis [2] due to its potent antiresorptive as well as possible bone-forming (anabolic) activities.

Numerous clinical studies have found gallium to have antineoplastic activity, particularly in some lymphomas [6], urothelial carcinoma [7], and nonsquamous cell cervical carcinoma [8]. As the antiproliferative properties of gallium extend to some micro-organisms, gallium has been suggested as a potential antibiotic, particularly for some intracellular infections such as tuberculosis [9], and as an antiretroviral agent, particularly against human immunodeficiency virus [10].

An orally active gallium compound was sought as a more convenient, comfortable, safe, and less costly alternative to parenterally administered gallium; in addition, such a compound could be used for chronic, widespread diseases such as postmenopausal osteoporosis. Gallium is absorbed very poorly when orally administered as salts such as the chloride or nitrate [11-13], due in part to hydrolysis that produces low-solubility polymerized gallium oxide hydroxides in the gastrointestinal fluids. In the animal and clinical studies reported here, gallium maltolate, tris(3-hydroxy-2-methyl-4*H*-pyran-4-onato)gallium (GaM), is found to provide oral gallium absorption at least several times higher than from gallium salts. This high oral bioavailability permits therapeutically useful blood gallium levels to be achieved without the need to orally administer large and possibly irritating or toxic doses of drug. In addition, the oral administration of gallium has been proposed to selectively increase gallium uptake by tumors and to reduce renal toxicity relative to parenteral administration [12].

Gallium maltolate is a coordination complex of a trivalent gallium ion with three deprotonated maltol (maltolate) groups (Figure 1). Maltol (2-methyl-3-hydroxy-4*H*-pyran-4-one) is produced by some plants and is commonly formed when sugars are heated: it is largely responsible for the scent of cotton candy and contributes significantly to the fragrance of many cakes, cookies, and candies. Its ability to provide à "fresh-baked" fragrance and to enhance sweet flavors has led to its extensive use as a food additive [14]. Maltol loses its hydroxyl proton at neutral to basic pH levels, forming the maltolate anion; this anionic molecule forms a strong bidentate chelate with gallium, as well as with iron, zinc, aluminum, vanadium, and many other metals (e.g.[15-19]).

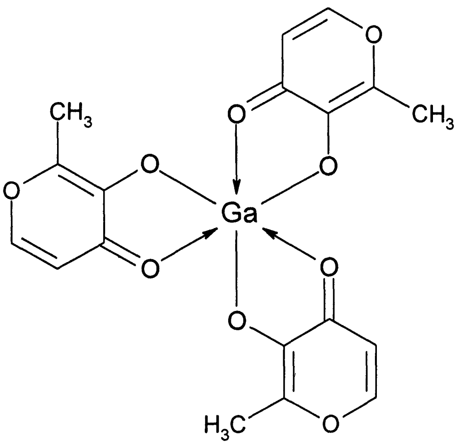

**Figure 1. Structural formula of gallium maltolate.**

### MATERIALS AND METHODS

### Synthesis of gallium maltolate

Gallium maltolate was synthesized by Regis Technologies, Morton Grove, Illinois. The compound was made by mixing aqueous solutions of maltol (0.5 M) and gallium nitrate nonohydrate ($Ga(NO_3)_3 \cdot 9H_2O$; 1 M) in proportions that produced a slight excess of maltol relative to a 3:1 molar ratio of maltol to gallium. The pH of the resulting solution was raised to approximately 7 by the gradual addition of solid sodium carbonate. The solution was heated to approximately 65°C for two hours and then cooled to 5 - 9°C. The resulting precipitate of white to light beige crystalline powder was predominately GaM plus several percent of excess maltol and residual sodium nitrates and carbonates. The powder was washed with water and then ethanol to remove impurities; it was then refluxed in ethanol, precipitated at 40-50°C, washed with ethanol, and dried.

### Physical, chemical, and crystallographic characterization

All chemicals other than GaM were ACS reagents obtained from Aldrich (Milwaukee, WI). Ultraviolet spectra of solutions, recorded between 190 and 400 nm, were obtained on a Cary 3 spectrophotometer. Infrared spectra between 400 and 4000 $cm^{-1}$ were obtained using KBr pellets on a BioRad Excalibur FTIR spectrophotometer. Inductively coupled plasma (ICP) spectrographic analyses for Ga, Na, and Cl, CNH analysis, and Karl-Fischer analysis for water were performed by Magellan Laboratories (North Carolina, USA). Density measurements were made using a Jolly balance with toluene. The octanol partition coefficient was measured (in duplicate) at 25°C by adding 0.22 g GaM to a beaker containing 20 mL water and 20 mL 1-octanol; the contents were stirred vigorously for 45 min then centrifuged for

30 sec to separate the solutions; 7.5 mL of each solution (water, 1-octanol) were evaporated at 50°C for 18 h, the evaporates were weighed, and the ratio of the weight of GaM precipitated from 1-octanol to that from water was determined. Stability of a dilute aqueous solution ($2.5 \times 10^{-5}$ M) as a function of pH was studied by measuring the absorbance at the 303 nm peak at pH values from 1.5 to 10 (at intervals of 0.5), using HCl and $Na_2CO_3$ to control pH values. Powder X-ray diffraction analysis was performed on a Siemens D-5000 diffractometer (Cu K$\alpha$ radiation, curved graphite diffracted beam monochromator, 40 kV, 35 mA).

For X-ray structural determination, a tabular crystal of gallium maltolate, approximately $130 \times 70 \times 20$ $\mu$m, was grown by adding 5 mL diethyl ether to an equal volume of a saturated ethanol solution of gallium maltolate at 25°C, then refrigerating at 4°C for 12 days. Single crystal X-ray diffraction analysis on this crystal was performed at the University of Colorado at Boulder using a Siemens SMART CCD diffractometer [Mo K$\alpha$ radiation ($\lambda$ = 0.71073 Å), graphite monochromator, using 5260 reflections (3255 independent) with intensity (I)>10$\sigma$ (I), at 158(2) K]. Data were corrected for Lorentz and polarization effects, and an absorption correction using redundant reflections was applied [20]. Data collection was 99.3% to 0.75 Å with a mean I/$\sigma$ of 1.09 in the shell between 0.80 and 0.75 Å; data above 0.84 Å were discarded during refinement due to poor intensity statistics. Centrosymmetric space group Pbca was uniquely chosen from systematic absences in the intensity data. Weighting was done according to: calc $w^{-1} = [\sigma^2(F_o^2)+(0.0279P)^2+3.9894P]$, where $P = (F_o^2+2F_c^2)/3$. The non-hydrogen structure was solved using direct methods (SHELXS-97 [21]). Refinement was of $F^2$ against all reflections.

**Dog bioavailability study**

Young adult male beagle dogs, 8 – 11 Kg each, were given either (1) intraduodenal gallium maltolate solution (10 mg/mL) (n=2), (2) oral gallium maltolate (n=2), (3) intraduodenal gallium nitrate solution (10 mg/mL) (n=2), or (4) intravenous citrated gallium nitrate (Ganite™) (25 mg/mL $GaNO_3$, 28.75 mg/mL sodium citrate dihydrate) (n=1) to roughly estimate relative bioavailabilities. Intraduodenal dosing was performed by first anesthetizing the dogs with an intravenous injection of I mL diazepam (5 mg/mL) and 0.7 mL ketamine (100 mg/mL) followed by intratracheal administration of halothane (2.5% in 1.5 L $O_2$). A flexible plastic tube was then guided through the mouth, esophagus, stomach and pylorus with the aid of an endoscope such that the distal end rested in the proximal duodenum; the test solutions were then injected into the tube and the tube flushed with an additional 5 mL deionized and UV-treated water. The oral doses were administered by gently forcing the dogs to swallow a gelatin capsule containing powdered gallium maltolate. The intravenous dose was administered as a bolus in the left saphenous vein. All dogs were dosed in the morning after fasting overnight and were fasted for an additional two hours after dosing. All the doses provided approximately 1.5 mg/Kg body weight of elemental Ga.

**Clinical study**

The clinical study was an open label, single-dose escalation study in normal volunteers; its primary objective was to evaluate the safety and tolerability of oral gallium maltolate in healthy subjects. A secondary objective was to determine pharmacokinetic parameters, including bioavailability, of orally administered gallium maltolate at four dose levels. Major inclusion criteria for the study were: normal, healthy males or females of age 21 through 60; weight at least 60 Kg and within 15% of ideal body weight; and values within normal ranges for vital signs, electrocardiogram, and laboratory tests. Major exclusion criteria were: presence of clinically significant history of disease, hypersensitivity to drugs, taking of any drugs within ten days prior to study entry, or participation in any investigational drug study within 30 days prior to study entry. The study was performed at Simbec Research Limited, Merthyr Tydfil, Wales, United Kingdom, in December 1997.

For human administration, gelatin capsules were filled with either 50 or 200 mg GaM plus the excipients Avicel® PH101 (microcrystalline cellulose), Kollidon 30 (polyvinyl pyrrolidone), Ac-Di-Sol® (internally cross-linked sodium carboxymethylcellulose), and stearic acid. The capsules were administered in a single dose. Four dosages were studied: 100 mg, 200 mg, 300 mg, and 500 mg. As gallium maltolate contains 15.67 weight percent Ga, these doses contain, respectively, 15.7, 31.3, 47.0, and 78.4 mg of Ga. Each dosage was administered to three subjects; the subjects were between the ages of 27 and 51, and all the subjects were male except for one female in the 300 mg group and one in the 500 mg group. The health of each subject was closely monitored during the study through daily (for four days) measurements of vital signs, blood chemistry, CBC with differentials and platelets, fecal occult blood, creatinine, and BUN, plus urinalysis with microscopic evaluation; on the fourth day following dosing, these tests were supplemented with a physical examination and 12-lead electrocardiogram, and all of these tests were repeated at 14±3 days post-dosing.

A 5 mL blood sample was taken from each subject at 0, 0.167, 0.33, 0.67, 1, 2, 3, 5, 8, 12, 16, 20, 24, 32, 40, 48, 60, 72, 84, 240, and 408 h following dosing. The blood samples were clotted within an hour of collection and serum collected by centrifugation. Urine was collected immediately pre-dose and over the intervals 0-4, 4-8, 8-12, 12-24, 24-48, and 48-72 h post-dosing. The serum and urine samples were analyzed for gallium concentration by Elemental Research, Inc. (N. Vancouver, British Columbia, Canada) using a validated inductively coupled plasma/mass spectrometry method.

This study of the safety, tolerance, and pharmacokinetics of single dose gallium maltolate was approved by the Simbec Research Ltd local research ethics committee following review of published clinical data on parenteral gallium nitrate and unpublished toxicological data on gallium maltolate orally administered to rats and dogs.

**Pharmacokinetic analysis**

Pharmacokinetic modeling was performed using the program WinNonLin 1.1 (Scientific Consultants, Inc., Apex, North Carolina, U.S.A./PharSight, Mountain View, California, U.S.A., 1995). One-, two-, and three-compartment models were considered. The statistically very small samples for the dog data justified only limited analysis.

To compare with our clinical results on oral gallium maltolate, data on intravenously administered gallium were obtained from the study of Kelsen et al. [22]. Serum gallium concentrations were reported by these authors for three cancer patients who received rapid intravenous doses of citrate-chelated gallium nitrate. One patient experienced renal failure and another had been treated immediately prior to gallium administration with cisplatin, adriamycin, and cyclophosphamide. The remaining patient (with malignant melanoma) had normal renal function and was not reported to have been heavily treated with toxic compounds immediately before gallium administration, and so was chosen as an example in the present study. This patient received 700 mg/M$^2$ of gallium nitrate. Assuming a surface area of 1.75 M$^2$, this dose is equivalent to approximately 1225 mg $Ga(NO_3)_3$. As $Ga(NO_3)_3$ contains 27.26 wt. percent Ga, a dose of 1225 mg contains approximately 334 mg Ga. The patient had a blood sample drawn prior to gallium administration, and other samples drawn at 0.083, 0.167, 0.25, 0.5, 1, 2, 4, 24, 48, and 104 hours following rapid intravenous administration of the gallium. The blood samples were centrifuged and the plasma analyzed for gallium concentration using flameless atomic absorption spectrometry.

To compare our clinical results with those for an orally administered gallium salt, pharmacokinetic data from a study by Collery et al. [12] were used. In their study a single oral dose of 800 mg $GaCl_3$ (containing 317 mg Ga) was administered to eighteen cancer patients; blood samples were drawn at intervals up to 96 h following dosing, and serum Ga concentrations were determined by atomic absorption spectrometry.

### RESULTS

**Physical properties, chemistry, and crystallography**

<u>Color</u>: White to light beige, becoming light yellow-brown upon exposure to air for more than approximately 48 h.

<u>Chemical analysis (weight percent)</u>: Ga 15.82, C 48.50, H 3.44, Na 0.0002, Cl <0.0001 (theoretical for $C_{18}H_{15}O_9Ga$: Ga 15.67, C 48.58, H 3.40). Karl-Fischer water content: 0.3 weight percent.

Solubility: 24(2) mM [10.7(9) mg/mL] in water at 25° C.
Octanol:water partition coefficient: 0.41(8) at 25° C.
Density: 1.56(5) g/cm$^3$ at 25° C.
Infrared spectroscopy: The major absorbance bands are presented in Table 1, and a representative spectrum is shown in Figure 2.
Ultraviolet spectroscopy: Broad absorption peaks occur (in order of intensity) at approximately 217, 303, and 250 nm.
Stability in dilute aqueous solution (2.5 × 10$^{-5}$ M) as a function of pH: The gallium maltolate complex appeared at least 98% undissociated over the pH range from approximately 5.5 to 8, with increased dissociation at higher and lower pH values.
Powder X-ray diffraction: The diffraction pattern corresponded to that previously reported [23], with no diffraction peaks observed for phases other than gallium maltolate.

**Table 1. Infrared absorption data.**

| Assignment[a] | cm$^{-1}$ | | Assignment | cm$^{-1}$ | Assignment | cm$^{-1}$ |
|---|---|---|---|---|---|---|
| νC–H (ring) | 3070 | m[b] | νC=C | 1610 | σC–H[c] | 1045 |
| νC–H ($CH_3$) | 2910 | m | νC=C | 1510 | σC–H[d] | 925 |
| νC=O | 1570 | | νC=C | 1465 | σC–H[d] | 850 |

[a]ν = vibration; σ = bending deformation. [b]Moderate intensity (others are strong or very strong). [c]In plane. [d]Out of plane.

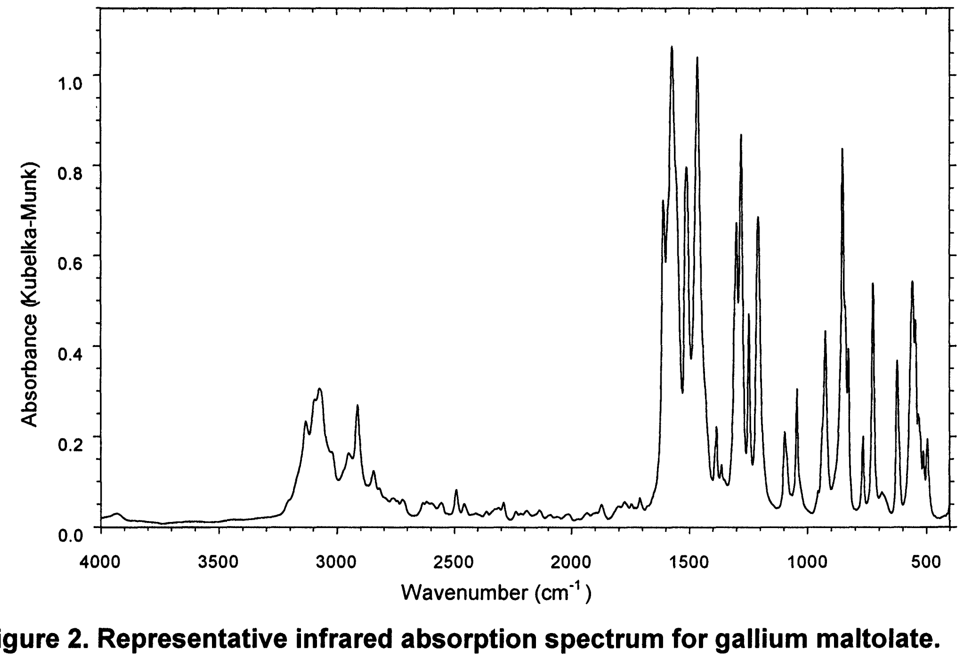

**Figure 2. Representative infrared absorption spectrum for gallium maltolate.**

Molecular and crystal structure: The structure of the GaM molecule based on the single-crystal X-ray diffraction study is shown in Figure 3, and the atomic and unit cell parameters of the crystal structure are presented in Tables 2 - 4. The structure is essentially the same as that for aluminum maltolate [16]. Refinement of both the Ga and Al maltolate structures indicates disorder of one of the three maltolate ligands. This disorder is manifested as two possible orientations of the ligand, which are nearly coplanar and are mirror images of each other. Close inspection of the environment around the disordered ligand reveals that out of the four possible pairings of this ligand with its inversion-related nearest neighbor, three result in reasonable interatomic distances. These three pairings contain a total

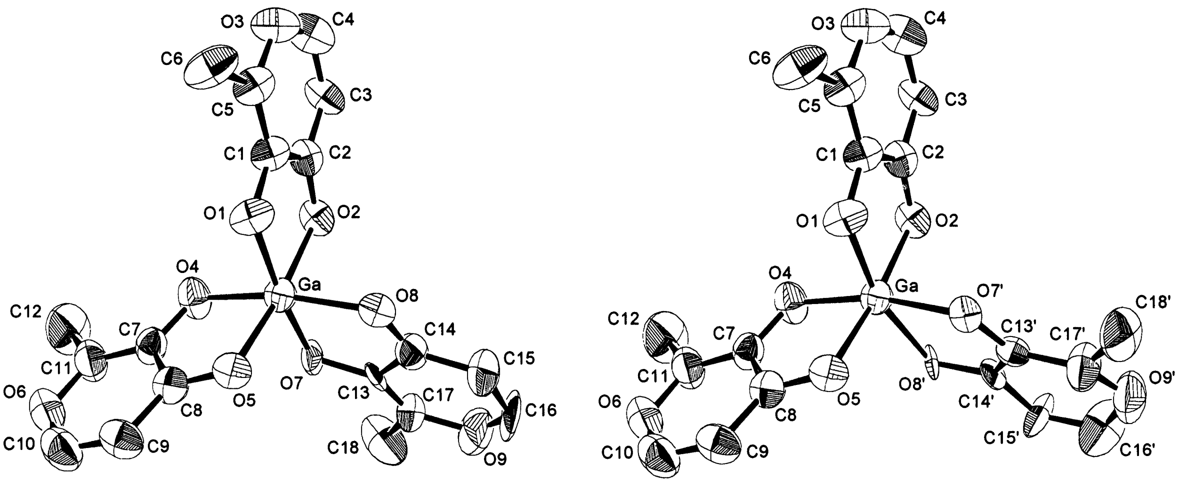

**Figure 3. Molecular structure of gallium maltolate derived from single-crystal X-ray diffraction study. The two observed isomers are shown, which differ only in the atomic positions for the lower-right ligand group; the minor components of this disordered ligand group are indicated by the " ' " symbol.**

of four ligands in one orientation and two ligands in the other orientation; the expected proportions of the two possible orientations (based strictly on probability) are thus 0.67 and 0.33. Refined occupancy values are 0.59(1) and 0.41(1); their difference from the expected values is likely due to small energy differences among the various pairs of permitted orientations. A calculated powder X-ray diffraction pattern using the refined structural data agrees very well with experimentally derived data [23]. The structural data are deposited with the Cambridge Crystallographic Data Centre (CCDC 138594).

**Table 2. Crystal data and refinement fit parameters for gallium maltolate.**

| | | | |
|---|---|---|---|
| Formula | $Ga(C_6H_5O_3)_3$ | Z (formula units/cell) | 8 |
| Formula mass | 445.02 | Density (g/cm$^3$) (calc.) (at 158 K) | 1.598 |
| Space group | *Pbca* | Density (g/cm$^3$) (obs.) (at 302 K) | 1.56(5) |
| *a* (Å) | 16.675(3) | Absorption coefficient (mm$^{-1}$) | 1.537 |
| *b* (Å) | 12.034(2) | Final R1[a] [I>2s(I)] | 0.0494 |
| *c* (Å) | 18.435(2) | Final wR2[b] [I>2s(I)] | 0.0830 |
| Cell volume (Å$^3$) | 3699.3(10) | Goodness-of-fit[c] on F$^2$ | 1.101 |

a) $R1 = \frac{\Sigma\left||F_o| - |F_c|\right|}{\Sigma|F_o|}$ b) $wR2 = \sqrt{\frac{\Sigma[w(F_o^2 - F_c^2)^2]}{\Sigma[w(F_o^2)^2]}}$ c) $GoF = S = \sqrt{\frac{\Sigma[w(F_o^2 - F_c^2)^2]}{(M-N)}}$ where M is the number of reflections and N is the number of parameters refined

### Dog bioavailability study

Results of the dog bioavailability study are summarized in Figure 4 and Table 5. The i.v. data were modeled using two compartments whereas the other data were well modeled using a single compartment. Absorption of Ga into blood plasma from intraduodenal or oral GaM was several times higher than from intraduodenal Ga nitrate in healthy young beagle dogs.

### Table 3. Atomic coordinates and isotropic displacement parameters for Ga maltolate.

| | x | y | z | $U_{eq}$ | Occupancy |
|---|---|---|---|---|---|
| Ga | 0.13208(3) | 0.22805(4) | 0.28342(3) | 0.04020(16) | 1 |
| O(1) | 0.05911(18) | 0.1533(2) | 0.21741(17) | 0.0501(8) | 1 |
| O(2) | 0.05118(17) | 0.3514(2) | 0.27684(18) | 0.0466(8) | 1 |
| O(3) | -0.12424(19) | 0.2594(3) | 0.13938(19) | 0.0609(9) | 1 |
| O(4) | 0.19997(19) | 0.2933(2) | 0.20950(18) | 0.0541(9) | 1 |
| O(5) | 0.21095(18) | 0.1025(2) | 0.27886(18) | 0.0459(8) | 1 |
| O(6) | 0.3860(2) | 0.1877(4) | 0.1382(2) | 0.0725(11) | 1 |
| O(7) | 0.1942(13) | 0.3221(18) | 0.3496(9) | 0.043(4) | 0.588(7) |
| O(8) | 0.0802(10) | 0.1743(9) | 0.3731(8) | 0.039(3) | 0.588(7) |
| O(9) | 0.1733(6) | 0.3428(11) | 0.5427(5) | 0.107(4) | 0.588(7) |
| C(1) | -0.0029(3) | 0.2177(4) | 0.2007(2) | 0.0442(12) | 1 |
| C(2) | -0.0063(3) | 0.3253(4) | 0.2344(3) | 0.0435(12) | 1 |
| C(3) | -0.0714(3) | 0.3952(4) | 0.2181(3) | 0.0534(13) | 1 |
| C(4) | -0.1280(3) | 0.3592(4) | 0.1727(3) | 0.0662(15) | 1 |
| C(5) | -0.0614(3) | 0.1891(4) | 0.1531(3) | 0.0514(13) | 1 |
| C(6) | -0.0669(3) | 0.0833(4) | 0.1117(3) | 0.0700(17) | 1 |
| C(7) | 0.2631(3) | 0.2293(4) | 0.1964(2) | 0.0458(12) | 1 |
| C(8) | 0.2672(3) | 0.1254(4) | 0.2343(3) | 0.0442(12) | 1 |
| C(9) | 0.3331(3) | 0.0557(4) | 0.2197(3) | 0.0580(13) | 1 |
| C(10) | 0.3894(3) | 0.0903(5) | 0.1729(3) | 0.0703(17) | 1 |
| C(11) | 0.3230(3) | 0.2566(5) | 0.1494(3) | 0.0632(15) | 1 |
| C(12) | 0.3291(4) | 0.3615(5) | 0.1075(4) | 0.099(2) | 1 |
| C(13) | 0.1711(12) | 0.304(2) | 0.4168(8) | 0.039(4) | 0.588(7) |
| C(14) | 0.1088(16) | 0.2242(19) | 0.4288(8) | 0.049(5) | 0.588(7) |
| C(15) | 0.0836(11) | 0.2041(16) | 0.5009(7) | 0.091(6) | 0.588(7) |
| C(16) | 0.1197(12) | 0.259(2) | 0.5545(8) | 0.135(12) | 0.588(7) |
| C(17) | 0.1992(8) | 0.3651(12) | 0.4744(7) | 0.068(4) | 0.588(7) |
| C(18) | 0.2635(6) | 0.4492(10) | 0.4728(6) | 0.111(5) | 0.588(7) |
| O(7') | 0.0832(15) | 0.1372(12) | 0.3667(11) | 0.033(4) | 0.412(7) |
| O(8') | 0.1816(16) | 0.3104(19) | 0.3683(11) | 0.029(4) | 0.412(7) |
| O(9') | 0.0844(9) | 0.2116(15) | 0.5548(8) | 0.094(5) | 0.412(7) |
| C(13') | 0.101(2) | 0.187(2) | 0.4287(10) | 0.035(6) | 0.412(7) |
| C(14') | 0.1517(19) | 0.281(3) | 0.4287(10) | 0.035(6) | 0.412(7) |
| C(15') | 0.1766(13) | 0.3297(17) | 0.4955(9) | 0.062(5) | 0.412(7) |
| C(16') | 0.1441(18) | 0.287(2) | 0.5556(10) | 0.088(10) | 0.412(7) |
| C(17') | 0.0709(16) | 0.1500(18) | 0.4938(10) | 0.069(7) | 0.412(7) |
| C(18') | 0.0072(8) | 0.0682(12) | 0.5007(8) | 0.083(5) | 0.412(7) |

Atoms marked with ( ' ) are minor components of the disordered ligand group. $U_{eq}$ (isotropic displacement parameter in $Å^2$) is defined as 1/3 the trace of the orthogonalized $U_{ij}$ tensor.

### Table 4. Bond lengths and angles surrounding Ga.

**Bond Lengths (Å)**

| | | | |
|---|---|---|---|
| Ga-O(4) | 1.938(3) | Ga-O(1) | 1.942(3) |
| Ga-O(7) | 1.960(19) | Ga-O(8) | 1.975(17) |
| Ga-O(5) | 2.004(3) | Ga-O(2) | 2.010(3) |
| Ga-O(8') | 2.03(2) | Ga-O(7') | 2.05(2) |

**Bond Angles (degrees)**

| | | | |
|---|---|---|---|
| O(4)-Ga-O(1) | 96.49(14) | O(4)-Ga-O(7) | 84.0(4) |
| O(1)-Ga-O(7) | 171.4(7) | O(4)-Ga-O(8) | 167.8(4) |
| O(1)-Ga-O(8) | 95.6(4) | O(7)-Ga-O(8) | 84.2(4) |
| O(4)-Ga-O(5) | 83.83(13) | O(1)-Ga-O(5) | 92.04(12) |
| O(7)-Ga-O(5) | 96.6(7) | O(8)-Ga-O(5) | 94.4(4) |
| O(4)-Ga-O(2) | 92.88(13) | O(1)-Ga-O(2) | 83.34(12) |
| O(7)-Ga-O(2) | 88.0(8) | O(8)-Ga-O(2) | 89.9(4) |
| O(5)-Ga-O(2) | 174.01(13) | O(4)-Ga-O(8') | 96.1(5) |
| O(1)-Ga-O(8') | 164.8(7) | O(7)-Ga-O(8') | 12.1(7) |
| O(8)-Ga-O(8') | 72.1(7) | O(5)-Ga-O(8') | 97.7(8) |
| O(2)-Ga-O(8') | 87.6(8) | O(4)-Ga-O(7') | 166.8(6) |
| O(1)-Ga-O(7') | 88.5(5) | O(7)-Ga-O(7') | 93.0(7) |
| O(8)-Ga-O(7') | 13.0(6) | O(5)-Ga-O(7') | 83.7(6) |
| O(2)-Ga-O(7') | 99.9(6) | O(8')-Ga-O(7') | 81.1(6) |
| C(1)-O(1)-Ga | 111.2(3) | C(2)-O(2)-Ga | 111.1(3) |
| C(7)-O(4)-Ga | 110.9(3) | C(8)-O(5)-Ga | 110.3(3) |
| C(13)-O(7)-Ga | 109.4(11) | C(14)-O(8)-Ga | 110.8(11) |
| C(13')-O(7')-Ga | 108.6(12) | C(14')-O(8')-Ga | 112.5(14) |

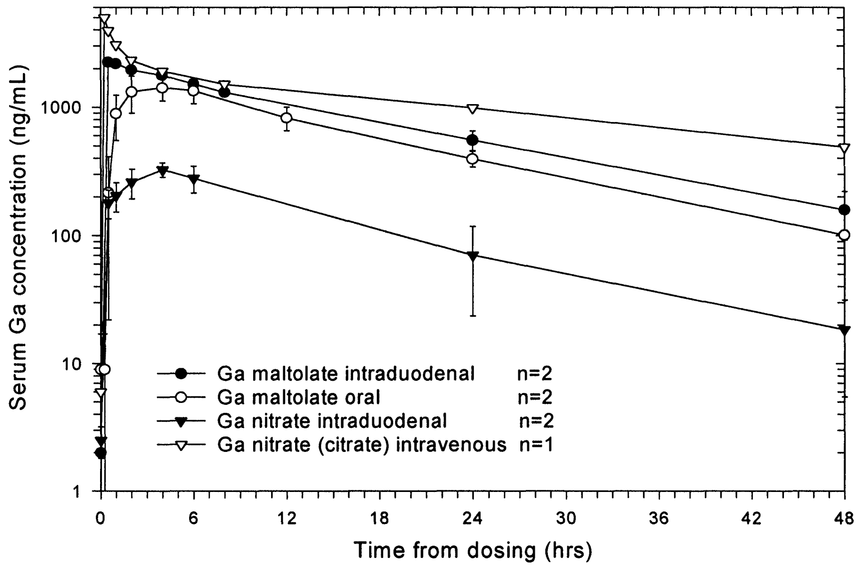

**Figure 4. Serum gallium concentrations as a function of time following administration of 1.5 mg/Kg Ga to young male beagle dogs as intraduodenal Ga maltolate or Ga nitrate, oral Ga maltolate, or intravenous Ga nitrate.**

**Table 5. Estimated pharmacokinetic and fitting parameters for 1.5 mg/Kg Ga administered to young male beagle dogs as intraduodenal Ga maltolate or Ga nitrate, oral Ga maltolate, or intravenous Ga nitrate. Standard errors are in parentheses.**

| | Ga maltolate intraduodenal | Ga maltolate oral | Ga nitrate intraduodenal | Ganite™ intravenous |
|---|---|---|---|---|
| $AUC_{0-\infty}$ (μg h/mL) | 37(2) | 27(1) | 5.6(1.0) | 68(7) |
| F ($AUC_{0-\infty}$) | 0.54 | 0.40 | 0.08 | [1.00] |
| α half life (h) | | | | 0.50(0.06) |
| β half life (h) | 10.7(0.7) | 10.4(0.7) | 9.9(2.4) | 21.3(2.9) |
| $K_{01}$ half life (h) | 0.08(5) | 0.64(7) | 0.8(0.2) | |
| $C_{max}$ (μg/mL) | 2.20(0.04) | 1.47(0.03) | 0.31(0.02) | 6.1(0.3) |
| $T_{max}$ (h) | 0.5(0.3) | 3.1(0.2) | 3.1(0.4) | [0] |
| V (L) | | | | 2.5(0.1) |
| $V_{ss}$ (L) | | | | 6.5(0.3) |
| CL (L/h) | | | | 0.22(0.02) |
| S | 0.00305 | 0.00654 | 0.02880 | 0.02966 |
| Correlation (r) | 0.9977 | 0.9985 | 0.9913 | 0.9909 |

Abbreviations are defined in Table 6.

### Clinical study

Gallium maltolate, at the doses studied, was very well tolerated by all the subjects, with no reports of serious treatment-related adverse events. Three of the twelve subjects reported possible drug-related adverse events: one subject in the 100 mg dose group reported a brief mild headache, and two subjects in the 200 mg group reported moderate coughs; these events all resolved without medical intervention.

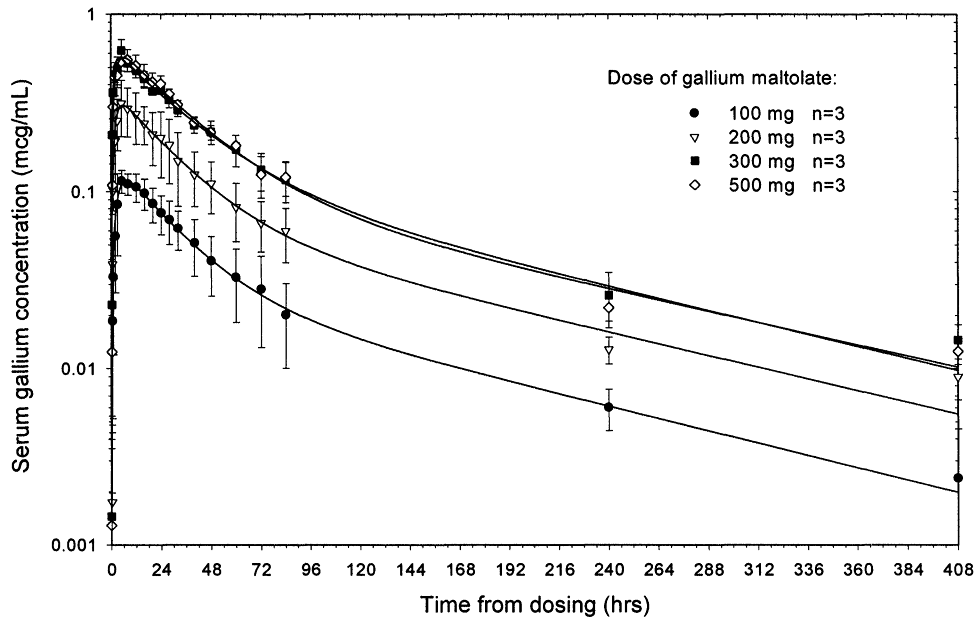

**Figure 5. Serum gallium concentrations as a function of time following administration of a single oral dose of gallium maltolate to healthy human subjects, with standard deviations. Curves represent 2-compartment (2-phase elimination) models.**

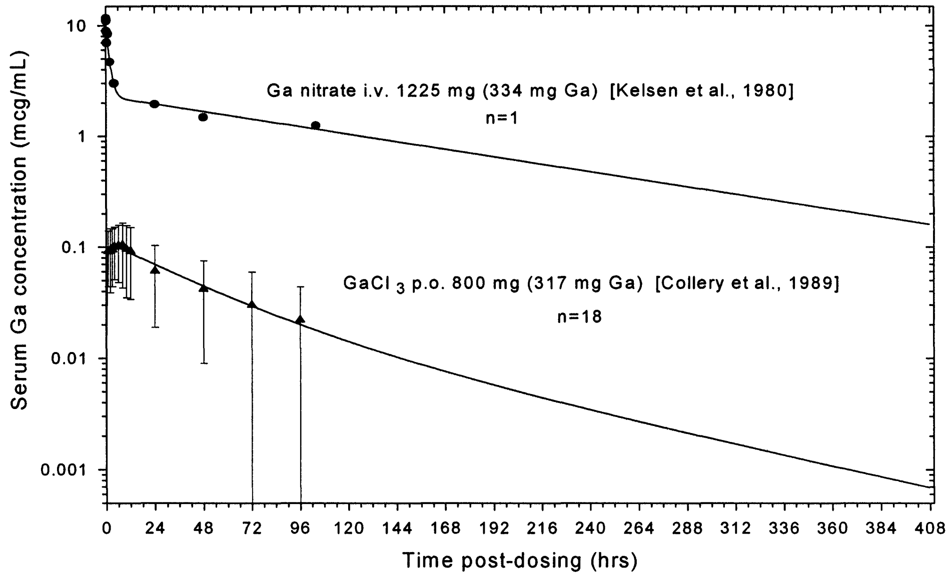

**Figure 6. Serum gallium concentrations as a function of time following a single rapid intravenous dose of about 1225 mg gallium nitrate (334 mg Ga) (Kelsen et al., 1980) or a single oral dose of 800 mg gallium chloride (317 mg Ga), with standard deviations. Curves represent 2-compartment (2-phase elimination) models.**

The data for oral GaM are plotted for each dose group in Figure 5. Data for i.v. GaN and oral $GaCl_3$ are plotted in Figure 6. Calculated curves based on two-compartment pharmacokinetic models are shown in both these figures. Cumulative urinary excretion for each dose group is shown in Figure 7. Estimated

**Table 6. Estimated pharmacokinetic and fitting parameters for orally administered gallium maltolate (serum gallium data), calculated using a two-phase elimination (first order) model. Standard errors are in parentheses.**

| Dose | 100 mg [15.7 mg Ga] | 200 mg [31.3 mg Ga] | 300 mg [47.0 mg Ga] | 500 mg [78.4 mg Ga] |
|---|---|---|---|---|
| $\alpha$ half life (h) | 17.3(3.2) | 18.4(2.5) | 21.3(7.7) | 21.4(8.0) |
| $\beta$ half life (h) | 104(51) | 109(46) | 107(117) | 115.0(148.6) |
| $K_{01}$ half life (h) | 2.0(0.2) | 1.4(0.1) | 0.8(0.1) | 1.4(0.2) |
| $K_{10}$ half life (h) | 34.9(5.2) | 36.4(4.5) | 39.9(11.3) | 37.8(11.7) |
| $K_{12}$ half life (h) | 53(16) | 58(14) | 69(41) | 77(51) |
| $K_{21}$ half life (h) | 53(29) | 53(20) | 58(67) | 63(103) |
| $AUC_{0-\infty}$ (μg h/mL) | 7.377 (1.034) | 19.265(2.296) | 36.305(10.031) | 36.763(10.920) |
| $T_{max}$ (h) | 7.8(0.3) | 6.3(0.2) | 4.3(0.4) | 6.4(0.5) |
| $C_{max}$ (μg/mL) | 0.115(0.002) | 0.306(0.004) | 0.566(0.016) | 0.569(0.017) |
| Lag time (h) | 0.32(0.05) | 0.42(0.04) | 0.24(0.06) | 0.20(0.08) |
| V/F (L) | 107.1(4.4) | 85.4(2.3) | 74.4(3.5) | 116.4(7.1) |
| F ($AUC_{0-\infty}$; Eq. 1) | 0.36 | 0.47 | 0.57 | 0.34 |
| F (V=29.5 L) | 0.28 | 0.35 | 0.40 | 0.25 |
| Ae (mg) | 0.35(0.14) | 0.66(0.28) | 1.11(0.08) | 1.37(0.52) |
| FAe | 0.022(0.001) | 0.021(0.001) | 0.023(0.000) | 0.018(0.001) |
| S | 0.00305 | 0.00654 | 0.02880 | 0.02966 |
| Correlation (r) | 0.9977 | 0.9985 | 0.9913 | 0.9909 |

**Abbreviations for Tables 4, 5 and 6:** **Ae**: Cumulative urinary excretion of gallium in 72h; **$AUC_{0-\infty}$**: Area under the serum level - time curve from time of dosing to infinite time; **$C_{max}$**: Maximum serum Ga concentration; **CL**: Total body clearance; **F**: Fraction of administered dose that is absorbed; **FAe**: Cumulative urinary excretion as fraction of administered dose; **$K_{xy}$**: Rate constants for transfer of Ga from compartment x to y, where for x or y, 0=external, 1=central, 2=second, and 3=third compartment; **r**: Correlation coefficient; **S**: Standard error of estimate; **$T_{max}$**: Time at which $C_{max}$ occurs; **V**: Volume of distribution of central compartment; **$V_{ss}$**: Volume of distribution at steady state; $\alpha,\beta,\gamma$: Rate constants for elimination phases.

pharmacokinetic parameters for oral GaM based on a two-compartment model are presented in Table 6; estimated pharmacokinetic parameters for i.v. GaN based on two- and three-compartment models are presented in Table 7.

### DISCUSSION

Semilogarithmic plots of the clinical serum Ga data (Figures 5,6) show that single compartment, first-order models (straight lines) are inadequate to model the oral GaM, oral $GaCl_3$, or i.v. GaN data, and that multi-compartment models are required. A two-compartment model provides a good fit to the oral GaM data, though the latest data point is generally underestimated (Figure 5). Although a three-compartment model will allow the latest data point to be better fit, use of such a model could not be justified without further information on gallium distribution and metabolism that support the existence and nature of the compartments, or without data points at later times that support the presence of a third elimination phase.

**Table 7. Estimated pharmacokinetic and fitting parameters for citrate-chelated gallium nitrate administered by rapid intravenous infusion (serum gallium data) (Kelsen et al., 1980), calculated using two-phase and three-phase elimination (first order) models. Standard errors are in parentheses.**

| | Dose: 1225 mg [334 mg Ga] | |
|---|---|---|
| | 2-Phase Model | 3-Phase Model |
| $\alpha$ half life (h) | 1.1(0.5) | 0.9(0.8) |
| $\beta$ half life (h) | 106.3(198.8) | 11.9(128.7) |
| $\gamma$ half life (h) | | 294(7174) |
| $K_{10}$ half life (h) | 22.4(31.4) | 43.2(790.1) |
| $K_{12}$ half life (h) | 1.5(0.5) | 1.5(2.8) |
| $K_{13}$ half life (h) | | 7(43) |
| $K_{21}$ half life(h) | 5.3(5.3) | 2.9(7.5) |
| $K_{31}$ half life (h) | | 25(367) |
| $AUC_{0-\infty}$ (μg h/mL) | 365(506) | 710(13003) |
| $C_{max}$ (μg/mL) | 11.3(0.8) | 11.4(1.2) |
| CL (L/h) | 0.9(1.3) | 0.4(11.6) |
| V (L) | 29.6(2.5) | 29.3(3.0) |
| $V_{ss}$ (L) | 135(87) | 189(1241) |
| S | 1.1184 | 1.3592 |
| Correlation (r) | 0.9736 | 0.9740 |

Similarly, the i.v. GaN data may be better fit using a three-compartment than a two-compartment model, but the lack of data beyond only 104 h makes the use of a three-compartment model highly speculative and results in very high fitting errors (Table 7). There appears, however, to be some justification for adding a third elimination phase in this case, due to the very rapid initial Ga elimination (half life ≈ 1 hr) that is not observed for orally administered GaM (Table 6).

It is noted that when ionic gallium is administered rapidly into the bloodstream, much of it is not able to bind immediately to transferrin (the predominate carrier of serum Ga at equilibrium); instead, significant amounts of anionic gallate ($Ga(OH)_4^-$) are formed [1,24]. Gallate, as a small charged molecule, is expected to be rapidly excreted by the kidneys. It is thus likely that the initial rapid elimination phase observed only with i.v. GaN is due at least in part to the rapid urinary excretion of gallate. Because Ga from oral GaM is absorbed more slowly into the bloodstream (absorption half life 2 - 3.9h, likely by the endogenous route for oral iron absorption onto serum transferrin), significant amounts of serum gallate are not expected to form. It therefore appears reasonable on biochemical grounds that the data for i.v. administration would require an additional rapid elimination phase not required for the oral administration data.

The hypothesis that a high proportion of plasma gallate relative to transferrin-bound gallium results following rapid i.v. GaN administration, whereas little if any plasma gallate results following oral GaM administration, is supported by the urinary excretion data. Cumulative Ga excreted in the urine following oral administration of GaM amounts to approximately 2% of the administered dose 72 h after dosing (Figure 7, Table 6). Inspection of the urinary excretion curves (Figure 7) shows that although

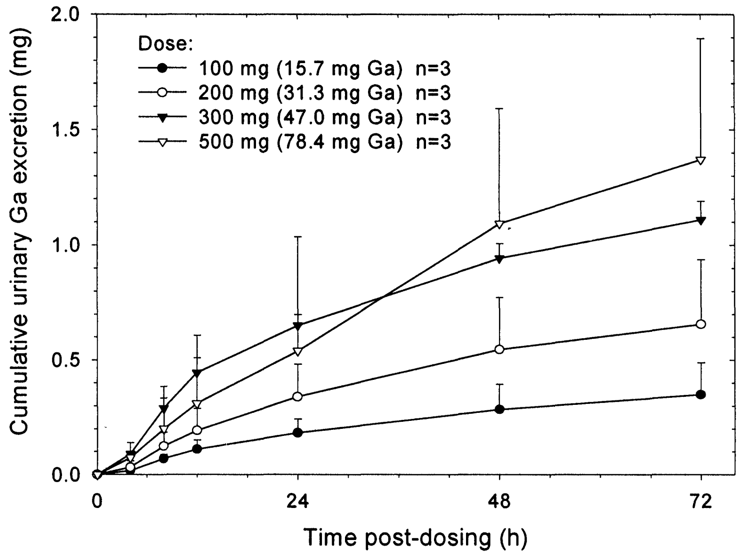

**Figure 7. Cumulative urinary gallium excretion as a function of time following administration of a single oral dose of gallium maltolate to healthy human subjects, with standard deviations.**

Ga excretion clearly appears to continue past 72 h, the decreasing slopes of the curves suggest that the ultimate cumulative excretion values are unlikely to be more than two or three times the measured 2% value. In contrast, Kelsen et al. [22] reported that, for a single rapidly administered i.v. dose of GaN, approximately 49-94% of the administered Ga dose was excreted in urine after only 24 h. This rapid and high urinary Ga excretion is most likely due to gallate excretion, whereas the low urinary Ga excretion from oral GaM is likely due to nearly all the Ga being protein-bound. The excretory route for this protein-bound Ga is not known, but may, as with some other metals, be fecal via bile. As previously discussed [1], high renal gallate levels may be responsible for the renal toxicity that was occasionally observed following high dose, bolus i.v. administration of gallium nitrate. If, as postulated, very little serum gallate results following oral GaM administration, then renal toxicity would not be expected. In fact, none of the subjects who took oral GaM showed any change in renal function, as measured by serum creatinine clearance, serum urea, blood urea nitrogen (BUN) clearance, urinary $\beta_2$-microglobulin, and complete urinalysis including microscopic examination.

The similarity of serum Ga data for the 300 mg and 500 mg groups (Table 6, Figure 5) suggests the possibility of saturation in absorption capacity above 300 mg. Examination of the data for individual subjects, however, suggests that the similarity in data for the two groups is likely due in large part to individual variations within statistically very small groups. This hypothesis is supported by the observation that estimates of $AUC_{0-\infty}$ and $C_{max}$ for oral GaM at the 100 and 500 mg dose levels (Table 6) indicate a roughly linear increase in blood serum levels and total absorption.

Oral bioavailability was first estimated by comparing the $AUC_{0-\infty}$ values calculated for oral GaM with the calculated $AUC_{0-\infty}$ value for i.v. GaN (representing 100% bioavailability). Assuming linear kinetics with dose, and assuming that i.v. GaN is eliminated in a similar manner to oral GaM (not an entirely valid assumption, as previously discussed), oral bioavailability (F) can be calculated by:

$$F = (AUC_{0-\infty})_{oral} / (AUC_{0-\infty})_{i.v} \times (Dose_{i.v.} / Dose_{oral}) \qquad \text{Eq. 1}$$

Using the two-phase elimination model, the F values derived by this method range from 0.34 to 0.57 (Table 6).

The oral bioavailability was also estimated by using the calculated V/F values. As the volume of distribution (V) cannot be derived independently from the oral GaM data, an estimate derived from the i.v. GaN data was used (bearing in mind that the differing plasma speciation and elimination of Ga derived from i.v. GaN and oral GaM may reduce the validity of the method). The estimated value of V for i.v. GaN is nearly the same for both the two-phase and three-phase elimination models: approximately 29.5 L (Table 7). If this quantity is substituted in the derived V/F values in the two-phase elimination model for oral GaM (Table 6), then F ranges from 0.25 to 0.40.

The derived value for the volume of distribution appears reasonable based on the known biochemistry of gallium. Nearly all plasma gallium at equilibrium, up to a gallium concentration of about 50 $\mu$M (3.5 $\mu$g/mL), is bound to the iron transport protein transferrin [1]. Transferrin is known to pass readily throughout the bloodstream, extracellular fluid, and intracellular fluid [25,26]. A value for the volume of distribution of approximately 25 - 30 L (as estimated here) is, in fact, within the range expected for a compound that occurs through the bloodstream, extracellular fluid, and intracellular fluid in an adult [27].

For orally administered $GaCl_3$, using the data and pharmacokinetic parameters supplied by Collery et al. [12], the gallium bioavailability is approximately 0.016 using the estimated $AUC_{0-\infty}$ value [6.394(5.352)$\mu$g h/mL], and approximately 0.010 using the estimated value for V/F [2862(297)L].

The mechanisms whereby gallium maltolate enhances the gastrointestinal absorption of gallium relative to that from gallium salts are not known. One likely mechanism is inhibition of hydrolysis: GaM maintains gallium in a stable, soluble, uncharged chelate at intestinal pH levels, preventing the formation of insoluble oxide-hydroxide polymers. In addition, as gallium maltolate is both hydrophilic and lipophilic (octanol partition coefficient 0.41(8)), it likely has high solubility in the intestinal mucus, facilitating gallium transit through the mucus layer, further permitting absorption. It is noted that the analogous iron compound, ferric maltolate, significantly enhances iron absorption in rats [28] and humans [29-31], and readily traverses the mucus layer and intestinal wall [28,32]. Barrand et al. [33] concluded that ferric maltolate dissociates before entry into the blood, with the dissociated iron following its endogenous uptake mechanism, ending up predominately on plasma transferrin. The dissociated maltol, based upon observations in dogs [34], rats [35], and humans (Hider, as referred to by Barrand et al. [33]), is conjugated in the small intestine (and, if it reaches the circulation, in the liver) with glucuronide or sulfate; the conjugate is then rapidly eliminated in the urine. Further studies will explore whether gallium maltolate displays similar behaviors. The exceptionally high intestinal metal bioavailability observed for both gallium maltolate and ferric maltolate suggests that a receptor-mediated absorption mechanism may exist; the existence and nature of this proposed mechanism are also under investigation.

### CONCLUSIONS

1. Gallium maltolate, in single doses of 100, 200, 300, and 500 mg, is very well tolerated by healthy human subjects.

2. The estimated oral bioavailability of gallium in humans from GaM is approximately 25 to 57%. This compares to an estimated 1 to 2% oral bioavailability from the salt gallium chloride. Dependence of bioavailability on dose for GaM was not observed over the dose range studied.

3. The dominant elimination half-life for gallium from blood plasma for oral GaM is approximately 17 to 21h, suggesting the potential for once-per-day dosing.

4. Approximately 2% of gallium from orally administered GaM is excreted in the urine within 72h following dosing. This compares to approximately 49-94% of an administered dose excreted within 24h following the rapid i.v. administration of citrate-chelated gallium nitrate [22]. The large difference is hypothesized to result from the formation of significant gallate [$Ga(OH)_4^-$] in blood plasma following the rapid i.v. administration of gallium nitrate. Gallate, as a small, charged molecule, will be rapidly excreted by the kidneys. In contrast, gallium orally absorbed from GaM is expected to follow the typical pathway for orally absorbed iron into plasma, with nearly all ending up bound to transferrin. This protein-bound gallium is not expected to have high urinary excretion; it may be excreted into the feces via bile.

5. The apparent nearly complete binding of gallium to plasma transferrin following oral administration of GaM appears to greatly reduce the potential for renal toxicity when compared to i.v. Ga nitrate. In addition, antineoplastic efficacy is expected to be enhanced, as most cancer cells are highly avid for transferrin-bound Ga relative to other forms of Ga [36].

6. Gallium maltolate appears to be a promising pharmaceutical compound for the oral administration of gallium.

### ACKNOWLEDGMENTS

We thank Dr. Gail L. Brown, Ms. Georgina Kilfoil, and Dr. Neil Gesundheit for their essential assistance in designing the clinical trial. We also thank Dr. Chozo Mitoma (SRI International, Menlo Park, CA USA) and Mr. Raffi Mikaelian (ITR Laboratories, Montreal, Canada) for their valuable assistance with the animal studies.

### REFERENCES

1. Bernstein LR (1998) *Pharmacol Rev* **50**:665-682.
2. Warrell RP Jr. (1995) *Handbook of Metal-Ligand Interactions in Biological Fluids, Bioinorganic Medicine* (Berthon G, ed) v 2, pp 1253-1265, Marcel Dekker, New York.
3. Bockman RS, Wilhelm F, Siris E, Singer F, Chausmer A, Bitton R, Kotler J, Bosco BJ, Eyre DR and Levenson D (1995) *J Clin Endocrinol Metab* **80**:595-602.
4. Warrell, RP Jr., Lovett D, Dilmanian FA, Schneider R and Heelan RT (1993) *J Clin Oncol* **11**:2443-2450.
5. Choy CG, Nieswizky R, Warrell RP Jr. and Michaeli I (1995) *Blood* **86**:831a.
6. Foster BJ, Clagett-Carr K, Hoth D and Leyland-Jones B (1986) *Cancer Treat Rep* **70**:1311-1319.
7. Einhorn LH, Roth BJ, Ansari R, Dreicer R, Gonin R and Loehrer PJ (1994) *J Clin Oncol* **12**:2271-2276.
8. Malfetano A, Blessing JA and Homesley D (1995) *Am J Clin Oncol* **18**:495-497.
9. Olakanmi O, Britigan BE and Schlesinger LS (1997) *J Invest Med* **45**:234A.
10. Stapleton JT, Klinzman D, Olakanmi O, Wuenschmann S, Schlesinger LS and Britigan BE (1999) Abstracts of the Interscience Conference on Antimicrobial Agents and Chemotherapy **39**:74.
11. Dudley HC and Levine MD (1949) *J Pharmacol Exper Ther* **50**:487-493.
12. Collery P, Millart H, Ferrand O, Jouet JB, Dubois JP, Barthes G, Pourny C, Pechery C, Choisy H, Cattan A, Dubois de Montreynaud JM and Etienne JC (1989) *Anticancer Res* **9**:353-356.
13. Ho DH, Lin JR, Brown NS and Newman RA (1990) *Eur J Pharmacol* **183**:1200.
14. LeBlanc DT and Akers HA (1989) *Food Technology* **43**:78-84.
15. Ahmet MT, Frampton CS and Silver J (1988) *J Chem Soc Dalton Trans* **1988**:1159-1163.
16. Finnegan MM, Rettig SJ and Orvig C (1985) *J Am Chem Soc* **108**:5033-5035.
17. Fryzuk MD, Jonker MJ and Rettig SJ (1997) *Chem Communications* **1997**:377-378.
18. Gérard C (1979) *Bull Soc Chim France* **1979**: I-451- I-456.
19. McNeill JH, Yuen VG, Hoveyda HR and Orvig C (1992) *J Med Chem* **35**:1489-1491.
20. Blessing RH (1995) *Acta Cryst* **A51**:33-38.
21. Sheldrick GM (1995) *SHELXTL, a program for crystal structure determination, version 5.03.* Siemens Analytical X-ray Instruments, Madison, Wisconsin.
22. Kelsen DP, Alcock N, Yeh S, Brown J and Young C (1980) *Cancer* **46**:2009-2013.

23. Bernstein LR (1995) *Powder Diffraction* **10:**140-142.
24. Weiner RE (1996) *Nucl Med Biol* **23:**745-751.
25. Huebers HA and Finch CA (1987) *Physiol Rev* **67:**520-582.
26. Brittenham GM (1991) *Hematology, Basic Principles and Practice* (Hoffman R, Benz EJ Jr., Shattil SJ, Furie B and Cohen HJ, eds) pp 327-349. Churchill Livingstone, New York.
27. Ritschel WA (1986) *Handbook of Basic Pharmacokinetics*. Drug Intelligence Publications, Hamilton, Illinois.
28. Barrand MA, Callingham BA and Hider RC (1987) *J Pharm Pharmacol* **39**:203-211.
29. Kelsey SM, Hider RC, Bloor JR, Blake DR, Gutteridge CN and Newland AC (1991) *J Clin Pharm Ther* **16:**117-122.
30. Maxton DG, Thompson RPH and Hider RC (1994) *Brit J Nutr* **71:**203-207.
31. Harvey RSJ, Reffitt DM, Doig LA, Meenan J, Ellis RD, Thompson RPH and Powell JJ (1998) *Aliment Pharmacol Ther* **12:**845-848.
32. Levey JA, Barrand MA, Callingham BA and Hider RC (1988) *Biochem Pharmacol* **37:**2051- 2057.
33. Barrand MA, Callingham BA, Dobbin P and Hider RC (1991) *Brit J Pharmacol* **102:**723-729.
34. Rennard HH (1971) *J Agr Food Chem* **19:**152-154.
35. Koster AS (1985) *Advances in Glucuronide Conjugation* (Matern S, Bock KW, and Gerock W eds) pp 177-195, HTP Press, Lancaster, Pennsylvania.
36. Chitambar CR and Seligman PA (1986) *J Clin Invest* **78:**1538-1546.

**Received: November 25, 1999 - Accepted: December 21, 1999 -**
**Received in revised camera-ready format: January 10, 2000**

